# Virucidal and Immunostimulating Activities of Monogalactosyl Diacylglyceride from *Coccomyxa* sp. KJ, a Green Microalga, against Murine Norovirus and Feline Calicivirus

**DOI:** 10.3390/md20020131

**Published:** 2022-02-10

**Authors:** Kyoko Hayashi, Satoko Komatsu, Hitoshi Kuno, Satomi Asai, Iori Matsuura, Vyankatesh Ramlu Kudkyal, Toshio Kawahara

**Affiliations:** 1 College of Life and Health Sciences, Chubu University, Kasugai 487-8501, Japan; kyhayashi@cronos.ocn.ne.jp (K.H.); rb21012-0952@sti.chubu.ac.jp (I.M.); 2 DENSO Corporation, Kariya 448-8661, Japan; satoko.komatsu.j8b@jp.denso.com (S.K.); hitoshi.kuno.j7c@jp.denso.com (H.K.); 3 School of Medicine, Tokai University, Isehara 259-1193, Japan; sa@is.icc.u-tokai.ac.jp; 4 Graduate School of Engineering, Chubu University, Kasugai 487-8501, Japan; te20801-7769@sti.chubu.ac.jp

**Keywords:** norovirus, monogalactosyl diacylglyceride, in vitro antiviral activity, mouse model, immunocompromised mice, neutralizing antibody

## Abstract

Human noroviruses are the most common pathogens causing acute gastroenteritis and may lead to more severe illnesses among immunosuppressed people, including elderly and organ transplant recipients. To date, there are no safe and effective vaccines or antiviral agents for norovirus infections. In the present study, we aimed to demonstrate the antiviral activity of monogalactosyl diacylglyceride (MGDG) isolated from a microalga, *Coccomyxa* sp. KJ, against murine norovirus (MNV) and feline calicivirus (FCV), the surrogates for human norovirus. MGDG showed virucidal activities against these viruses in a dose- and time-dependent manner—MGDG at 100 μg/mL reduced the infectivity of MNV and FCV to approximately 10% after 60 min incubation. In the animal experiments of MNV infection, intraoral administration of MGDG (1 mg/day) exerted a therapeutic effect by suppressing viral shedding in the feces and produced high neutralizing antibody titers in sera and feces. When MGDG was orally administered to immunocompromised mice treated with 5-fluorouracil, the compound exhibited earlier stopping of viral shedding and higher neutralizing antibody titers of sera than those in the control mice administered with distilled water. Thus, MGDG may offer a new therapeutic and prophylactic alternative against norovirus infections.

## 1. Introduction

Norovirus, belonging to the family Caliciviridae, is a non-enveloped virus with an icosahedral structure of 38-nm diameter. Its genome consists of single-stranded RNA of approximately 7.5 kb in length. Human noroviruses (HNoVs) are the most common viral cause of acute gastroenteritis, accounting for approximately 20% of diseases worldwide [1,2]. HNoV infection causes profuse vomiting and diarrhea, which are typically self-limiting. Although several types of candidate vaccines are being developed [3], there are no reports on approved safe and effective vaccines and anti-norovirus drugs. In the absence of vaccines and antiviral agents for HNoV, developing prophylactic or therapeutic measures against the virus remains an important issue.

Monogalactosyl diacylglyceride (MGDG) is a glycoglycerolipid found in vegetables, fruits, and grains [4]. As MGDG is consumed daily [5], its toxicity seems unproblematic. In addition, MGDGs from algae exhibit anti-tumor and anti-inflammatory activities [6,7]. Recently, it was found that MGDG isolated from a microalga, *Coccomyxa* sp. KJ (IPOD FERM BP-22254) displays a virucidal effect on herpes simplex virus type 2 (HSV-2) [8]. HSV-2, an enveloped virus, is a genital herpes pathogen. Morphological studies have suggested that MGDG can lyse the viral envelope as well as the viral capsid inside the envelope. Based on these findings, we expect that MGDG might also show inhibitory effects on noroviruses having the capsid on the outermost surface.

Owing to insufficient replication in the cell culture system for HNoVs, we used two cultivable strains of noroviruses, feline calicivirus (FCV) and murine norovirus (MNV), as surrogates for HNoVs in the present study. 

This study aimed to evaluate the potential of MGDG as a candidate for developing antiviral agents to treat HNoV infections. We assessed the in vitro virucidal activities of MGDG against FCV and MNV and its in vivo therapeutic effects in MNV-infected mice in both immunocompetent and immunocompromised states. In mouse models of MNV, the virus infects macrophages and dendritic cells, possibly in a manner dependent on microfold (M) cells associated with Peyer’s patches [9]. In MNV-infected mice, while the innate immune responses, including interferon production, are important for suppressing viral replication, initiating the adaptive immune response plays a key role in clearing viral infection and generating immunological memory to prevent reinfection [10]. The findings of this study offer a new therapeutic and prophylactic alternative against norovirus infections.

## 2. Results

### 2.1. In Vitro Antiviral Activity of MGDG

MGDG was evaluated for its antiviral potency against MNV and FCV, added immediately after viral infection. The values of selectivity indices calculated from CC_50_/EC_50_ were 1.1 ± 0.14 and 1.1 ± 0.035 for MNV and FCV, respectively (Table 1). In general, when a selectivity index is >10, a sample can be regarded as possessing antiviral activity. These results showed that MGDG had no marked anti-MNV and anti-FCV activities in vitro.

**Figure 1:**
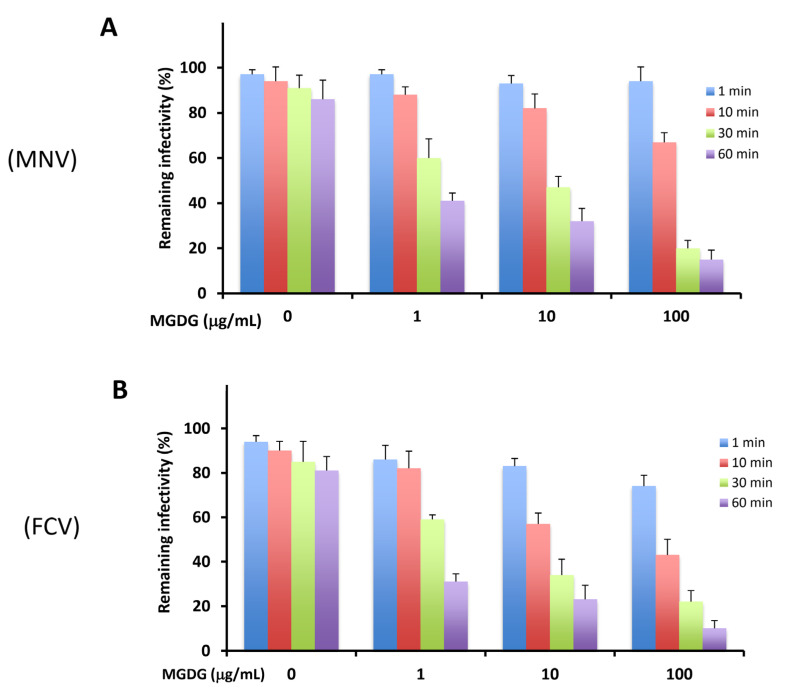
Virucidal activities of monogalactosyl diacylglyceride (MGDG) against murine norovirus (MNV) (**A**) and feline calicivirus (FCV) (**B**). Virus (2 × 10^5^ plaque-forming units (PFU)/mL) was mixed with an equal volume of 0, 1, 10, and 100 μg/mL MGDG and incubated for the indicated time at 37 °C. Results are expressed as the percentages of residual infectivity of MGDG-treated virus compared to the residual infectivity of the mock-treated virus control. Data are the means from independent duplicate assays.

As reported previously [8], MGDG affects the HSV from outside the host cell by directly inactivating virus particles released from infected cells. Therefore, we investigated whether MGDG reduces the infectivity of MNV and FCV due to the interaction between MGDG and virus particles. The virucidal activity of MGDG was assessed using an assay based on incubating the compound–virus mixture before calculating residual virus infectivity via a plaque assay. As shown in Figure 1, MGDG inactivated both MNVs and FCVs in a dose- and time-dependent manner.

**Figure 2:**
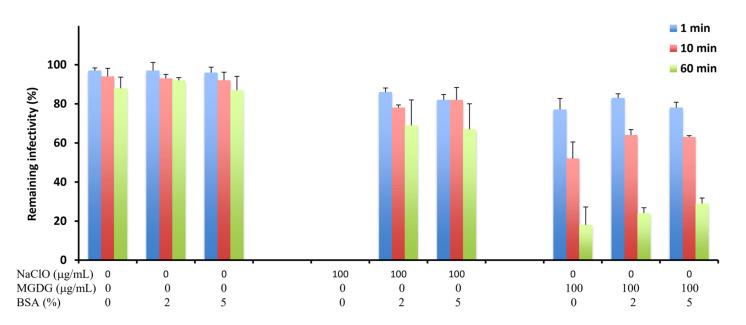
Virucidal activities of MGDG and sodium hypochlorite (NaClO) against FCV in the presence of bovine serum albumin (BSA). FCV (2 × 10^5^ PFU/mL) was mixed with an equal volume of 0 and 100 μg/mL MGDG or NaClO in the presence of 0%, 2%, and 5% BSA and incubated for the indicated time at 37 °C. Results are expressed as the percentages of residual infectivity of MGDG-treated virus compared to the residual infectivity of the mock-treated virus control. Data are the means from independent duplicate assays.

Furthermore, we assessed the effects of an organic substance, bovine serum albumin (BSA), on the virucidal activity of MGDG compared with that of NaClO, a potent disinfectant agent against norovirus. Incubation of FCV with 100 μg/mL NaClO in the absence of BSA, no infectious virus was detected within 1 min after incubation (Figure 2). However, the virucidal activity dropped remarkably in the presence of 2% or 5% BSA, with approximately 60% FCV infectivity remaining even after 60 min of incubation. In contrast, no significant decrease in the virucidal activity of 100 μg/mL MGDG was observed in the presence of BSA.

### 2.2. In Vivo Therapeutic Effects of MGDG on MNV Infection

**Figure 3:**
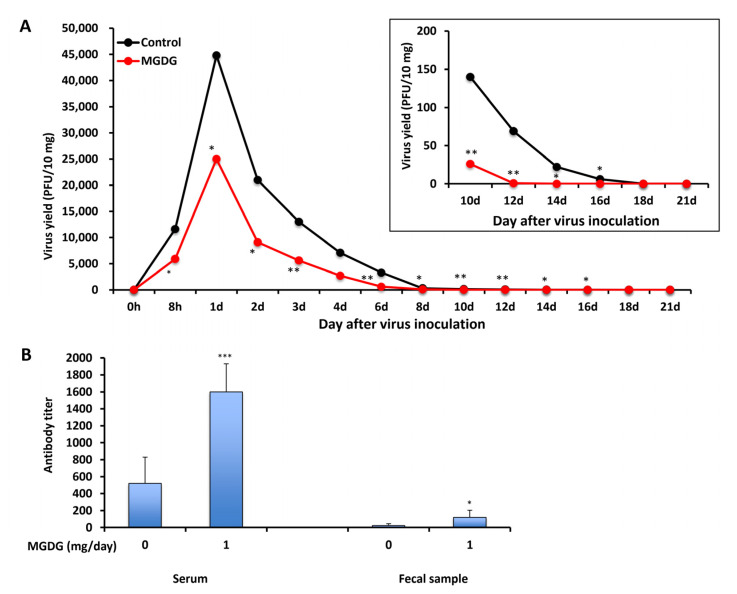
In vivo efficacy of MGDG. BALB/c mice (*n* = 3 per group) were inoculated with 1 × 10^6^ PFU of MNV, and 1 mg of MGDG/day was administered 1 h after infection until 21 d post-infection (p.i.). The control group was administered with distilled water. (**A**) Virus yields in the feces collected from 0 h to 21 d p.i.; (**B**) neutralizing antibody titers in sera and feces at 21 d p.i. * *p* < 0.05; ** *p* < 0.01; *** *p* < 0.001 vs. control.

BALB/c mice were infected perorally with 1 × 10^6^ plaque-forming units (PFU) of MNV, and MGDG was administered 1 h after infection until 21 d post-infection (p.i.). All mice survived the experiment, and no diarrhea or weight loss was observed during the 21 d experimental period. The viral titers in the feces were determined by plaque assay using RAW 267.4 cells. MNV was detected in the feces of both control and MGDG-administered groups as early as 8 h p.i. However, MGDG-administered mice showed significantly reduced titers of MNV from 8 h to 16 d p.i., compared to those in the control group (Figure 3A). Viral shedding was stopped at 18 and 14 d p.i. in the control and MGDG-administered groups, respectively.

In an attempt to elucidate whether orally administering MGDG could stimulate systemic and local immunoresponses in MNV-infected animals, virus-neutralizing antibody titers in sera and feces were determined on day 21 p.i. (Figure 3B). The titer in serum was 1600 ± 330 in the MGDG-administered group, which was significantly higher than that in the control group (520 ± 310) (*p* < 0.001). In addition, MGDG produced higher antibody titers in feces (120 ± 84) than control (23 ± 21) (*p* < 0.05). 

### 2.3. In Vivo Effects of MGDG on MNV Infection in Immunocompromised Mice

**Figure 4:**
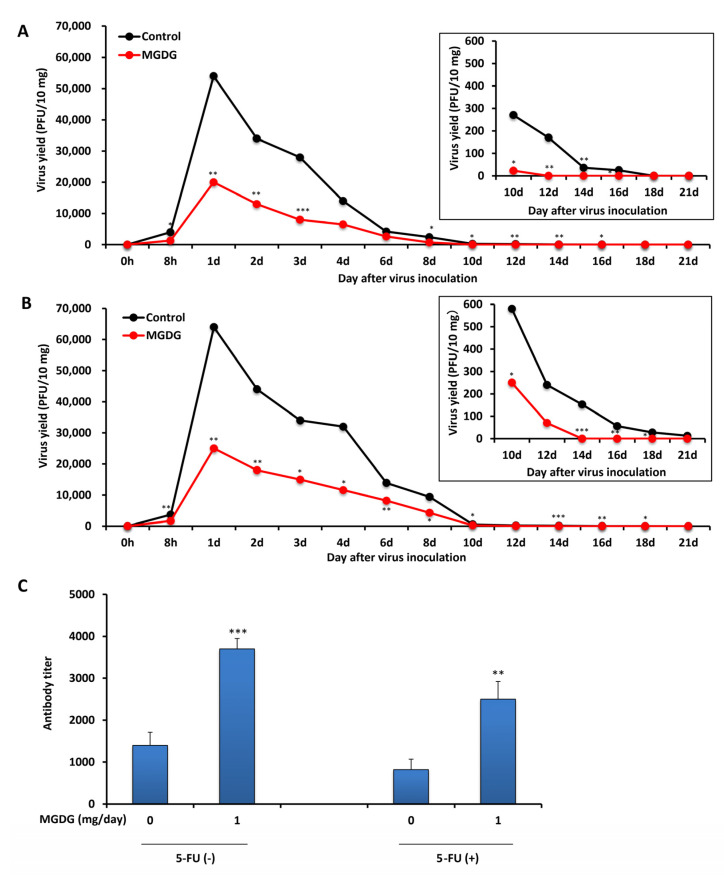
In vivo efficacy of MGDG in immunocompromised mice. BALB/c (*n* = 5 per group) inoculated perorally with 1 × 10^6^ PFU of MNV. A dose of 1 mg MGDG/day was administered from 7 d before viral infection until 21 d p.i. The control group was administered with distilled water. Fecal samples were collected from 0 h to 21 d p.i. (**A**) Virus yields of mice without 5-fluorouracil (5-FU) treatment in the feces; (**B**) virus yields of mice with 5-FU treatment in the feces; (**C**) neutralizing antibody titers in sera at 21 d p.i. in the group without 5-FU treatment [5-FU(−)] and the group with 5-FU treatment [5-FU(+)]. * *p* < 0.05; ** *p* < 0.01; *** *p* < 0.001 vs. control.

As shown in Figure 3, MGDG increased MNV-specific antibody production in immunocompetent mice. To evaluate this immunostimulatory effect in immunocompromised mice, the animals were treated with 5-fluorouracil (5-FU) to suppress the immune function. All immunocompetent mice without 5-FU treatment and immunocompromised mice with 5-FU treatment survived throughout the experiments of 21 d. The 5-FU-treated control mice showed prolonged viral shedding of >21 days p.i. (Figure 4B) compared with immunocompetent control mice (Figure 4A) in which viral shedding ceased at 18 d p.i. In MGDG-administered immunocompromised mice, prolonged viral shedding at 14 d p.i. (Figure 4B) was observed compared with MGDG-administered immunocompetent mice (Figure 4A) in which viral shedding ceased at 12 d p.i. MGDG administration significantly reduced and earlier stopping of viral shedding (Figure 4B) compared with immunocompromised control mice. The neutralizing antibody titers of sera were reduced by 5-FU treatment in the control and MGDG-administered mice by 59% and 68%, respectively (Figure 4C). The antibody production significantly increased in the MGDG group of both immunocompetent (*p* < 0.001) and immunocompromised mice (*p* < 0.01) compared with the corresponding control groups.

## 3. Discussion

Eom et al. reported that the extract of brown alga *Eisenia bicyclis* and its components (dieckol and phlorofucofuroeckol-A) exhibited in vitro anti-MNV with high selectivity indices [11]. The study of in vitro inhibitory effects of fucoidans (sulfated polysaccharides) obtained from brown algae *Laminaria japonica* and *Undaria pinnatifida* against MNV and FCV have demonstrated that fucoidans exerted no marked virucidal activity after 3 h of incubation with the viruses at a concentration of 100 μg/mL, and the log_10_ reduction in virus titers ranged from 0.23 to 0.52 [12]. In contrast, MGDG showed approximately 1.0 log_10_ reduction in FCV titer after 1 h of incubation at the same concentration (Figure 1B). Orally administered fucoidans also reduced the viral titers in the feces of mice. In addition, some natural compounds derived from plants exert anti-norovirus activities. Phenolic compounds such as quercetin show virucidal against MNV [13]. Allspice oil, lemongrass oil, and citral also produced marked in vitro inactivation of MNV [14]. The extract and some components obtained from *Lindera obtusiloba* leaf exerted mild virucidal activity against MNV [15]. In this study, MGDG, a natural product obtained from an alga, exhibited in vitro virucidal activity against MNV and FCV, in vivo suppressive effects on viral replication, and immunostimulating effects in MNV-infected animals.

In this study, MGDG demonstrated virucidal activity against non-enveloped viruses, FCV and MNV. Transmission electron microscopy images of MGDG-treated HSV-2 particles showed that MGDG treatment might cause morphological changes in not only the envelope but also the capsids of the virus particles, as judged from the reduction of their diameter. The MNV capsid plays a crucial role in binding to its receptor on host cells [16].

NaClO is effective against enveloped viruses and is recommended for disinfecting HNoV in Japan [17]; however, the presence of organic substances, such as BSA, quickly deactivates NaClO [17]. In this study, we showed that the virucidal activity of MGDG was maintained at a higher level even in the presence of BSA compared with the activity of NaClO (Figure 2).

In the in vivo study using the experimental animals, orally administered MGDG suppressed MNV excretion into the feces (Figure 3A) and elevated neutralizing antibody titers in the sera (Figure 3B). Considering that a certain level of virucidal effect of MGDG could be maintained even in the presence of organic substances (Figure 2), orally administered MGDG possibly exerted a certain degree of direct inactivation of inoculated or propagated MNV in the intestinal tract where organic substances coexist. The combination of these effects might have suppressed the viral replication and early clearance of the virus in MGDG-administered mice. Therefore, the effects of MGDG on immune function need further investigation.

As B-cell deficient mice incapable of producing norovirus-specific antibodies did not clear MNV, B cells are hypothesized to help clear the viral infection through antibody production [18]. Supporting this hypothesis, MGDG-treated mice showed high secretory antibody titers in the mucosa and early clearance of viral infection in the present study (Figure 3). Secretory IgA antibody in feces plays a crucial role in suppressing MNV replication in the intestinal mucosa. As Ward et al. [19] indicated, MNV inoculation was not associated with illness or death in experimental mice used in the present study.

Infectious gastroenteritis caused by HNoV is a common acute illness that is characteristically self-limiting. However, it may lead to more severe or protracted illness among young children, the elderly, and organ transplant recipients [20,21,22,23]. Immunosuppressed patients experience prolonged fecal HNoV shedding, shedding the viruses for months or even years after clearance of symptoms [24,25,26]. The immunocompromised state, which mimics the lowering of the immune function due to aging, stress, or medical intervention, can be experimentally produced in animals by treatment with some anti-cancer drugs, including 5-FU [27,28,29]. As reported previously, mice treated with 5-FU reduced the natural killer cell activity [30]. In the present study, we performed virological evaluation in MNV-inoculated mice injected with 5-FU. The shedding period of norovirus extended in an immunosuppressive state caused by 5-FU treatment in mice as well as in humans, as shown in Figure 4B. Oral administration of MGDG shortened the viral shedding period compared with the control mice, possibly due to the elevation of virus-specific antibody titers (Figure 4). 

In conclusion, this study is the first to report the suppression of viral replication and immunostimulation in MNV-infected animals by the galactolipid contained in an alga. Given that the MNV capsid plays a crucial role in binding to its receptor on host cells, this study suggests that MGDG treatment might affect the viral capsids and exert its virucidal activity by inhibiting the binding of the virus to host cells via the capsids. However, the exact mechanism of virucidal action exerted by MGDG needs to be confirmed in future studies.

## 4. Materials and Methods

### 4.1. Preparation of MGDG

MGDG was isolated from the ethanol extract of *Coccomyxa* sp. KJ, grown in an inorganic medium as described previously [31], following the bioassay-guided fractionation via column chromatography as described previously [8]. Hexane solution of MGDG was subjected to gas chromatography-mass spectrometry to determine its chemical structure [8]. 

### 4.2. Cells and Viruses

The FCV F4 strain used in the present study was isolated from a cat with signs of respiratory tract infection [32] and obtained from Dr. Tohya, Nihon University in Japan. Crandell Rees feline kidney (CRFK) cells obtained from Nihon University were used as host cells and cultured in Eagle’s minimal essential medium (MEM) supplemented with 5% fetal bovine serum (FBS) and antibiotics (100 U/mL of penicillin and 100 μg/mL of streptomycin; Nacalai Tesque, Inc., Kyoto, Japan). The viruses were plaque-titrated in CRFK cells with MEM containing 1% SeaPlaque agarose (Lonza, Rockland, ME, USA). The MNV S7-PP3 strain, genetically close to MNV3 [33], was isolated from mouse stools in Japan [34]. In this study, it was obtained from Dr. Tohya, Nihon University, Japan. This strain has been widely used in investigating the receptor for MNV [35] and tests for virucidal and antiviral substances [16,36]. The MNV S7-PP3 strain was propagated in RAW 264.7 cells (ATCC TIB-71). The cells were cultured in Dulbecco’s modified Eagle medium (DMEM) supplemented with 10% FBS and antibiotics. MNV was titrated by plaque assay in RAW 264.7 cells with DMEM containing 1.5% SeaPlaque agarose.

### 4.3. In Vitro Antiviral Assays

MGDG was dissolved in <0.5% dimethyl sulfoxide, which did not interfere with the growth of cells or viruses (data not shown). Control without MGDG contained 0.5% dimethyl sulfoxide. For cytotoxicity assays, CRFK and RAW 264.7 cells were seeded at a density of 1 × 10^4^ cells/well in 96-well plates. After 24 h incubation at 37 °C, the cells were treated with 0–1000 μg/mL MGDG at 37 °C for 72 h. Viable cells were counted using the trypan blue exclusion test, and the 50% cytotoxic concentration (CC_50_) was calculated from concentration-response curves. For antiviral assays, CRFK and RAW 264.7 cell monolayers in 48-well plates were infected with FCV and MNV, respectively, at 0.1 PFU/cell for 1 h at room temperature. The cell monolayers were washed with phosphate-buffered saline (PBS) and further incubated at 37 °C for 24 h in the presence of 0–1000 μg/mL MGDG. After fixation with a 10% formaldehyde solution for 2 h at room temperature, the cell monolayers were stained with trypan blue solution and subjected to plaque counting under the microscope [37]. Virus yields were determined using plaque assays, and the 50% effective concentration (EC_50_) was obtained from the concentration–response curves. The antiviral activities of MGDG against FCV and MNV were estimated using the selective index calculated from the CC_50_s and EC_50_s. Data are shown as the mean ± SD from independent duplicate assays. 

### 4.4. Virucidal Assay

We determined the direct inactivation of MNV and FCV by MGDG. The viruses (1 × 10^5^ PFU/mL) were incubated at 37 °C in the presence of 0, 1, 10, and 100 μg/mL MGDG. After 0, 1, 10, 30, and 60 min, 100 μL of 100-fold dilution of the mixture was added to host cell monolayers in 35-mm dishes for 1 h at room temperature. Then the medium for plaque titration mentioned above was added to the dishes. The plaque number at 0 h was considered 100%. The virucidal effects of MGDG on FCV were examined in the presence of an organic substance, BSA (Nacalai Tesque), and compared with those of NaClO, which is frequently used as a disinfectant.

### 4.5. Animals

Female BALB/c mice (5–6 weeks old) were purchased from Japan SLC (Shizuoka, Japan). All animal experiments were performed per the animal experimentation guidelines of Chubu University and approved by the Animal Care Committee at Chubu University. After blood collection, the mice were euthanized by anesthesia. No side effects, such as diarrhea, due to virus inoculation or drug administration were observed throughout the experiments.

### 4.6. Animal Experiments

To evaluate the therapeutic effect of MGDG, mice were perorally inoculated with MNV (1 × 10^6^ PFU/0.2 mL PBS) (*n* = 3 per group). MGDG was first dissolved in 100% ethanol at 20 mg/mL and then diluted with distilled water to 1 mg/0.4 mL, which contained 5% ethanol. MGDG treatment was initiated 1 h after infection with a dose of 1 mg/0.4 mL/day until 21 d p.i. by oral gavage. Control mice were administered with distilled water containing 5% ethanol. Stools were collected from each mouse during the experimental period. For fecal MNV titers, stool sample homogenates were centrifuged at 3000 rpm for 15 min, and the supernatant was collected as a fecal suspension. RAW 264.7 cell monolayers in a 24-well plate were infected at room temperature for 1 h with 100 μL of serial 10-fold dilutions of the fecal suspension for plaque titration. In another experiment, an immunocompromised group of mice was subcutaneously injected with 0.5 mg of 5-FU per treatment every alternate day from 7 d before virus inoculation (1 × 10^6^ PFU/0.2 mL PBS) until 21 d after inoculation. Both immunocompromised mice with 5-FU treatment (*n* = 5) and immunocompetent mice without 5-FU treatment (*n* = 5) were administered with 1 mg MGDG/day from 7 d before virus inoculation until 21 d after inoculation.

### 4.7. Assay for Neutralizing Antibody

Neutralizing anti-MNV antibody titers of the serum and feces were determined using a 50% plaque reduction assay. Approximately 2 × 10^3^ PFU/mL virus was mixed with an equal volume of serum or feces at 10 to 50,000 dilutions with PBS and incubated at 37 °C for 1 h. Each mixture was added at 100 μL/dish onto RAW 264.7 cell monolayers in 35-mm dishes for plaque assay. The neutralizing antibody titer was considered the highest dilution of the serum or feces, which reduced the plaque numbers by 50%, compared with the control containing PBS instead of serum or feces.

### 4.8. Statistical Analysis

Comparisons between two groups of animals were made using the Student’s *t*-test. The results with a *p*-value < 0.05 were considered statistically significant.

## Figures and Tables

**Table 1:** Anti-feline calicivirus (FCV) and anti-mouse norovirus (MNV) activities of monogalactosyl diacylglyceride.

Virus	Cytotoxicity	Antiviral Activity	Selectivity Index
	(CC_50_, μg/mL)	(EC_50_, μg/mL)	(CC_50_/EC_50_)
FCV	122 ± 11	117 ± 6.4	1.1 ± 0.035
MNV	195 ± 9.2	177 ± 11	1.1 ± 0.14

Each value represents the means ± SD from independent duplicate assays. Sample was added immediately after the viral infection.

## References

[1] Ahmed S.M., Hall A.J., Robinson A.E., Verhoef L., Premkumar P., Parashar U.D., Koopmans M., Lopman B.A. (2014). Global Prevalence of Norovirus in Cases of Gastroenteritis: A Systematic Review and Meta-analysis. Lancet Infect. Dis..

[2] Payne D.C., Vinjé J., Szilagyi P.G., Edwards K.M., Staat M.A., Weinberg G.A., Hall C.B., Chappell J., Bernstein D.I., Curns A.T. (2013). Norovirus and Medically Attended Gastroenteritis in US Children. N. Engl. J. Med..

[3] Treanor J.J., Atmar R.L., Frey S.E., Gormley R., Chen W.H., Ferreira J., Goodwin R., Borkowski A., Clemens R., Mendelman P.M. (2014). A Novel Intramuscular Bivalent Norovirus Virus-Like Particle Vaccine Candidate–Reactogenicity, Safety, and Immunogenicity in a phase 1 Trial in Healthy Adults. J. Infect. Dis..

[4] Kasai Y., Oshima K., Ikeda F., Abe J., Yoshimitsu Y., Harayama S. (2015). Construction of a Self-Cloning System in the Unicellular Green Alga *Pseudochoricystis ellipsoidea*. Biotechnol. Biofuels.

[5] Maeda N., Koka Y., Hada T., Yoshida H., Muzushina Y. (2013). Oral Administration of Monogalactosyl Diacylglycerol from Spinach Inhibits Colon Tumor Growth in Mice. Exp. Ther. Med..

[6] Morimoto T., Nagatsu A., Murakami N., Sakakibara J., Tokuda H., Nishino H., Iwashima A. (1995). Anti-Tumour-Promoting Glyceroglycolipids from the Green Alga, *Chlorella vulgaris*. Phytochemistry.

[7] Kikuchi H., Tsukitani Y., Manda T., Fujii T., Nakanishi H., Kobayashi M., Kitagawa I. (1982). Marine Natural Products. X. Pharmacologically Active Glycolipids from the Okinawan Marine Sponge *Phyllospongia foliascens* (Pallas). Chem. Pharm. Bull..

[8] Hayashi K., Lee J.B., Atsumi K., Kanazashi M., Shibayama T., Okamoto K., Kawahara T., Hayashi T. (2019). *In Vitro* and *In Vivo* Anti-Herpes Simplex Virus Activity of Monogalactosyl Diacylglyceride from *Coccomyxa sp. KJ* (IPod FERM BP-22254), a Green Microalga. PLoS ONE.

[9] Wobus C.E., Karst S.M., Thackray L.B., Chang K.O., Sosnovtsev S.V., Belliot G., Krug A., Mackenzie J.M., Green K.Y., Virgin H.W. (2004). Replication of Norovirus in Cell Culture Reveals a Tropism for Dendritic Cells and Macrophages. PLoS Biol..

[10] Newman K.L., Leon J.S. (2015). Norovirus Immunology: Of Mice and Mechanisms. Eur. J. Immunol..

[11] Eom S.H., Moon S.Y., Lee D.S., Kim H.J., Park K., Lee E.W., Kim T.H., Chung Y.H., Lee M.S., Kim Y.M. (2015). *In Vitro* Antiviral Activity of Dieckol and Phlorofucofuroeckol-A Isolated from Edible Brown Alga *Eisenia bicyclis* against Murine Norovirus. Algae.

[12] Kim H., Lim C.Y., Lee D.B., Seok J.H., Kim K.H., Chung M.S. (2020). Inhibitory Effects of *Laminaria Japonica* Fucoidans against Noroviruses. Viruses.

[13] Iloghalu U., Holmes B., Khatiwada J., Williams L.L. (2019). Selected Plant Extracts Show Antiviral Effects against Murine Norovirus Surrogate. Adv. Microbiol..

[14] Gilling D.H., Kitajima M., Torrey J.R., Bright K.R. (2014). Mechanisms of Antiviral Action of Plant Antimicrobials against Murine Norovirus. Appl. Environ. Microbiol..

[15] Solis-Sanchez D., Rivera-Piza A., Lee S., Kim J., Kim B., Choi J.B., Kim Y.W., Ko G.P., Song M.J., Lee S.J. (2020). Antiviral Effects of *Lindera obtusiloba* Leaf Extract on Murine norovirus-1 (MNV-1), a Human Norovirus Surrogate, and Potential Application to Model Foods. Antibiotics.

[16] Walker F.C., Hassan E., Peterson S.T., Rodgers R., Schriefer L.A., Thompson C.E., Li Y., Kalugotla G., Blum-Johnston C., Lawrence D. (2021). Norovirus Evolution in Immunodeficient Mice Reveals Potentiated Pathogenicity via a Single Nucleotide Change in the Viral Capsid. PLoS. Pathog..

[17] Aoyama T., Kudo T. (2021). Comparison of the Disinfecting Effect of Sodium Hypochlorite Aqueous Solution and Surfactant on Hospital Kitchen Hygiene Using Adenosine Triphosphate Swab Testing. PLoS ONE.

[18] Chachu K.A., Strong D.W., LoBue A.D., Wobus C.E., Baric R.S., Virgin H.W. (2008). Antibody Is Critical for the Clearance of Murine Norovirus Infection. J. Virol..

[19] Ward J.M., Wobus C.E., Thackray L.B., Erexson C.R., Faucette L.J., Belliot G., Barron E.L., Sosnovtsev S.V., Green K.Y. (2006). Pathology of Immunodeficient Mice with Naturally Occurring Murine Norovirus Infection. Toxicol. Pathol..

[20] Trivedi T.K., DeSalvo T., Lee L., Palumbo A., Moll M., Curns A., Hall A.J., Patel M., Parashar U.D., Lopman B.A. (2012). Hospitalization and Mortality Associated with Norovirus Outbreaks in Nursing Homes. J. Am. Med. Assoc..

[21] Schwartz S., Vergoulidou M., Schreier E., Loddenkemper C., Reinwald M., Schmidt-Hieber M., Flegel W.A., Thiel E., Schneider T. (2011). Norovirus Gastroenteritis Causes Severe and Lethal Complications after Chemotherapy and Hematopoietic Stem Cell Transplantation. Blood.

[22] Bagci S., Eis-Hübinger A.M., Yassin A.F., Simon A., Bartmann P., Franz A.R., Mueller A. (2010). Clinical Characteristics of Viral Intestinal Infection in Preterm and Term Neonates. Eur. J. Clin. Microbiol. Infect. Dis..

[23] Bok K., Green K.Y. (2012). Norovirus Gastroenteritis in Immunocompromised Patients. N. Engl. J. Med..

[24] Henke-Gendo C., Harste G., Juergens-Saathoff B., Mattner F., Deppe H., Heim A. (2009). New Real-Time PCR Detects Prolonged Norovirus Excretion in Highly Immunosuppressed Patients and Children. J. Clin. Microbiol..

[25] Ludwig A., Adams O., Laws H.J., Schroten H., Tenenbaum T. (2008). Quantitative Detection of Norovirus Excretion in Pediatric Patients with Cancer and Prolonged Gastroenteritis and Shedding of Norovirus. J. Med. Virol..

[26] Schorn R., Höhne M., Meerbach A., Bossart W., Wüthrich R.P., Schreier E., Müller N.J., Fehr T. (2010). Chronic Norovirus Infection after Kidney Transplantation: Molecular Evidence for Immune-Driven Viral Evolution. Clin. Infect. Dis..

[27] Umeda Y., Sakamoto A., Nakamura J., Ishitsuka H., Yagi Y. (1983). Thymosin Alpha 1 Restores NK-Cell Activity and Prevents Tumor Progression in Mice Immunosuppressed by Cytostatics or X-Rays. Cancer Immunol. Immunother..

[28] Rasi G., Silecchia G., Sinibaldi-Vallebona P., Spaziani E., Pierimarchi P., Sivilia M., Tremiterra S., Garaci E. (1994). Anti-Tumor Effect of Combined Treatment with Thymosin Alpha 1 and Interleukin-2 after 5-Fluorouracil in Liver Metastases from Colorectal Cancer in Rats. Int. J. Cancer.

[29] Rafique M., Adachi W. (1995). Effects of Intraportal Administration of Chemoimmunotherapeutic Agents on Natural Killer Cell Activity in the Rat Liver. J. Surg. Oncol..

[30] Hayashi K., Nakano T., Hashimoto M., Kanekiyo K., Hayashi T. (2008). Defensive Effects of a Fucoidan from Brown Alga *Undaria pinnatifida* against Herpes Simplex Virus Infection. Int. Immunopharmacol..

[31] Satoh A., Kato M., Yamato K., Ishibashi M., Sekiguchi H., Kurano N., Miyachi S. (2010). Characterization of the Lipid Accumulation in a New Microalgal Species, *Pseudochoricystis ellipsoidea* (Trebouxiophyceae). J. Jpn. Inst. Energy.

[32] Takahashi E., Konishi S., Ogata M. (1971). Studies on Cytopathogenic Viruses from Cats with Respiratory Infections. Jpn. J. Vet. Sci..

[33] Kitajima M., Oka T., Tohya Y., Katayama H., Takeda N., Katayama K. (2009). Development of a Broadly Reactive Nested Reverse Transcription-PCR Assay to Detect Murine Norovirus, and Investigation of the Prevalence of Murine Noroviruses in Laboratory Mice in Japan. Microbiol. Immunol..

[34] Kitagawa Y., Tohya Y., Ike F., Kajita A., Park S.J., Ishii Y., Kyuwa S., Yoshikawa Y. (2010). Indirect ELISA and Indirect Immunofluorescent Antibody Assay for Detecting the Antibody against Murine Norovirus S7 in Mice. Exp. Anim..

[35] Haga K., Fujimoto A., Takai-Todaka R., Miki M., Murakami K., Yokoyama M., Murata K., Nakanishi A., Katayama K. (2016). Functional Receptor Molecules CD300lf and CD300ld within the CD300 Family Enable Murine Norovirus to Infect Cells. Proc. Natl. Acad. Sci. USA.

[36] Kitajima M., Tohya Y., Matsubara K., Haramoto E., Utagawa E., Katayama H. (2010). Chlorine Inactivation of Human Norovirus, Murine Norovirus and Poliovirus in Drinking Water. Lett. Appl. Microbiol..

[37] Ito T., Hayashi K., Nishiguchi M., Hayashi T., Iimuna M. (2018). Resveratrol Oligomer C-Glucosides and Antiviral Resveratrol Tetramers Isolated from the Stem Bark of *Shorea uliginosa*. Phytochem. Lett..

